# Using Unsupervised Machine Learning to Identify Age- and Sex-Independent Severity Subgroups Among Patients with COVID-19: Observational Longitudinal Study

**DOI:** 10.2196/25988

**Published:** 2021-05-27

**Authors:** Julián Benito-León, Mª Dolores del Castillo, Alberto Estirado, Ritwik Ghosh, Souvik Dubey, J Ignacio Serrano

**Affiliations:** 1 Department of Neurology University Hospital “12 de Octubre” Madrid Spain; 2 Neural and Cognitive Engineering Group Center for Automation and Robotics CSIC-UPM Arganda del Rey Spain; 3 HM Hospitales Madrid Spain; 4 Department of General Medicine Burdwan Medical College and Hospital Burdwan India; 5 Department of Neuromedicine Bangur Institute of Neurosciences Kolkata India

**Keywords:** COVID-19, machine learning, outcome, severity, subgroup, emergency, detection, intervention, testing, data set, characterization

## Abstract

**Background:**

Early detection and intervention are the key factors for improving outcomes in patients with COVID-19.

**Objective:**

The objective of this observational longitudinal study was to identify nonoverlapping severity subgroups (ie, clusters) among patients with COVID-19, based exclusively on clinical data and standard laboratory tests obtained during patient assessment in the emergency department.

**Methods:**

We applied unsupervised machine learning to a data set of 853 patients with COVID-19 from the HM group of hospitals (HM Hospitales) in Madrid, Spain. Age and sex were not considered while building the clusters, as these variables could introduce biases in machine learning algorithms and raise ethical implications or enable discrimination in triage protocols.

**Results:**

From 850 clinical and laboratory variables, four tests—the serum levels of aspartate transaminase (AST), lactate dehydrogenase (LDH), C-reactive protein (CRP), and the number of neutrophils—were enough to segregate the entire patient pool into three separate clusters. Further, the percentage of monocytes and lymphocytes and the levels of alanine transaminase (ALT) distinguished cluster 3 patients from the other two clusters. The highest proportion of deceased patients; the highest levels of AST, ALT, LDH, and CRP; the highest number of neutrophils; and the lowest percentages of monocytes and lymphocytes characterized cluster 1. Cluster 2 included a lower proportion of deceased patients and intermediate levels of the previous laboratory tests. The lowest proportion of deceased patients; the lowest levels of AST, ALT, LDH, and CRP; the lowest number of neutrophils; and the highest percentages of monocytes and lymphocytes characterized cluster 3.

**Conclusions:**

A few standard laboratory tests, deemed available in all emergency departments, have shown good discriminative power for the characterization of severity subgroups among patients with COVID-19.

## Introduction

The COVID-19 pandemic has brought to light the scarcity of health care resources worldwide [1]. One of the main challenges faced by health care systems while tackling this pandemic is the lack of affordable, accurate, and simple information that can allow clinicians to predict the evolution of the patients’ disease sooner, upon admission to the hospital. This information might help clinicians to make early decisions regarding arrangement and organization of medical resources, as well as early interventions to improve the health outcomes of these patients.

The exhaustive and inefficiently structured amount of health data available does not permit parametric modeling in an easy way. To overcome this issue, machine learning techniques have recently been identified as promising tools in data analysis for individual class prediction, allowing us to deal with a great number of variables simultaneously and observe inherent disease-related patterns in the data [2].

Machine learning for health care is a key discipline aimed to translate large health data sets into operative knowledge in different medical fields [3-7]. The methods of this artificial intelligence paradigm can be classified as supervised or unsupervised, based on the underlying strategy used [8]. In inductive or supervised machine learning, the method builds a general class description of the target categories from a set of previously categorized examples [8]. In general, supervised learning methods are used to design classifiers from labeled samples that predict the class of an unseen new sample [8]. In the field of medicine, these methods have been applied to find prognostic and predictive biomarkers [9]. On the other hand, in unsupervised machine learning, the goal is to find the class or classes that cover the sample [8]. These methods permit the discovery of the underlying structure and relationships among unlabeled samples [8]. Unsupervised clustering techniques can obtain groups of samples so that the intrasimilarity within each group is maximized, while intersimilarity between groups is minimized [8]. They are usually applied in medicine to identify homogeneous groups of patients based on their medical records and relationships between clinical manifestations and therapeutic responses, or to detect sets of coexpressed genes, among other applications [10,11].

There are several research reports using COVID-19 data sets, which focus on predicting the patients’ mortality or severity by mainly using regression modeling from labeled clinical records [12-17]. Further, in a multicenter study, using supervised machine learning, a personalized COVID-19 mortality risk score for hospitalized patients upon admission has been proposed [18]; however, in that study [18], the reason for choosing only a subset of the recorded clinical variables to build their model was not explained. Therefore, the algorithm might have been biased, even by the expert’s knowledge. In all of these studies [12-18] and in a study based on cluster analysis [19], demographics, such as age and sex, were considered as key variables in their prediction models. By contrast, these variables were deliberately excluded from the training data set in this study, in which we used an unsupervised machine learning method for data handling.

Health agencies recommend that clinical decisions should be made based on an individual’s biological age rather than chronological age [20,21]. There are multiple physiological and molecular markers for estimation of biological aging that can predict life span [22]. Besides these markers, the heterogeneity of eating habits, physical and mental conditions, and therapeutics have an influence on the overall health state, making biological aging a heterogeneous process too.

Frailty and multi-morbidities, as measures of biological aging, have been found to be risk factors for mortality independent of chronological age in patients with COVID-19 [23]. New procedures for the therapeutic management of COVID-19 are required regardless chronological age [24].

Furthermore, reports about case-fatality rates for COVID-19 categorized by age groups could sentence elderly people not only to social exclusion but also to health care indifference. Considering the elderly population as a highly vulnerable group is a simple and negative stereotype that may even influence decision making in clinical resource management [25].

The prevalence and severity of COVID-19 also varies based on sex, whereby men experience higher mortality than women [26]. The severity of the disease implies that the person may need hospitalization, intensive care support, and mechanical ventilation. However, the medical treatments scheduled during hospitalization or a stay in intensive care are the same for every patient with a severe case of COVID-19, regardless age or sex [27].

Since chronological age as well as sex cannot be considered as pivotal aspects to determine an individual’s health status and resilience [28], these should not be key determinants for health care or resource allocation among people suffering from COVID-19. Therefore, predictive models based on intelligent data processing that take into account a patient’s age as a major determinant in health care access may be inappropriate and unethical [25].

Demographic variables (ie, age and sex) were not used in the previously published studies for building models on effective treatments based upon sex or age groups or for understanding sex or age differences [12-19]. These predictive models of severity and mortality risk for COVID-19 could be discriminating [29]. For example, consideration of the age of people in the emergency department might discriminate against older people (ie, ageism) regarding access to care, since the decision would be based purely upon the age of the patients rather than their health care needs [30].

The objective of this observational longitudinal study was to identify nonoverlapping severity subgroups (ie, clusters) among patients with COVID-19, using exclusive laboratory tests and clinical data obtained during the first medical contact in the emergency department, by means of unsupervised machine learning techniques. Age and sex were not taken into account to build the subgroups due to the ethical implications. For this purpose, we used the data set collected by the HM group of hospitals (HM Hospitales) in Madrid, Spain [31].

## Methods

### Data Set

This study is a longitudinal analysis of the data set collected by the HM group of hospitals in Madrid, Spain, in the context of the project Covid Data Save Lives [31]. The information from this data set comes from the electronic health records data system of the seven HM hospitals, located in the Community of Madrid in Spain [31]. This data set contains the anonymized records of 2310 patients, admitted to any of the seven HM hospitals, with a diagnosis of COVID-19 from March 1 to April 24, 2020. The data set includes different interactions within COVID-19 treatment processes, including detailed information on diagnoses, treatments, intensive care unit (ICU) admission, and discharge or death, among many other variables. The data set also includes diagnostic imaging and laboratory tests or records of previous medical care, if any. It also includes the drugs administered to each patient during admission (more than 60,000 records) with the dates corresponding to the first and last administration of each drug, which was identified by its brand name and classification in the Anatomical Therapeutic Chemical codes (ATC5/ATC7). Moreover, laboratory data are also included (398,884 records). Finally, the data set contains the records of the diagnostic and procedural information—coded according to the ICD-10 (International Statistical Classification of Diseases and Related Health Problems, 10th Revision) classification in its latest distributed version—for the patients referred, both for episodes of hospital admission (more than 1600) and for the emergencies (more than 1900) prior to those episodes, if any.

### Data Preprocessing

We collected the information for each patient identifier and compiled it into one record. This included age, sex, vital signs in the emergency department, and the need or lack of need of the ICU. COVID-19 symptoms, ICD-10 codes of previous and current conditions, as well as different laboratory tests performed in the emergency department were also recorded. We also calculated, for each patient, the duration in days of the hospital stay, including ICU admission and the days from hospitalization to ICU admission. We also considered the first laboratory tests obtained from the emergency department and grouped all of the ICD-10 codes under the first three characters (ie, first letter and two subsequent numbers) of the code to reduce the number of variables and provide generalization. We codified each ICD-10 feature for inclusion in one of the following groups: *present in emergency department admission*, *not present in emergency department admission*, or *developed during hospital stay*.

Only patients with a discharge reason of *death* or *recovered* were included in the analyses. The patients with a discharge reason of *transferred to another hospital* or *transferred back to the nursing home* (about 3.6% of the total data set) were excluded, since no additional information was available after they left the hospital. We only selected the records (ie, patients) with no missing values on clinical data and laboratory tests, which left a final sample of 853 (37.2% women) patients to be included in our analyses. The mean age of the sample was 67.2 (SD 15.7) years (range 21-106). Each patient had 850 variables in his or her record, including eight variables about demographics, hospitalization stay, and outcome measures; one variable about COVID-19 symptoms; 10 variables about vital signs (eg, temperature, heart rate, oxygen saturation, and systolic and diastolic blood pressure) in the emergency department; 29 laboratory tests from the emergency department (Table 1); 168 ICD-10 codes from the emergency department; and 634 ICD-10 codes during their hospital stay.

The final sample of 853 patients was similar to the excluded sample (n=1457) in terms of age (mean 67.2, SD 15.7 years, vs mean 67.1, SD 17.0 years; *F*_1,2308_=1.508; *P*=.22); discharge reason (selected deceased: 15.6% vs excluded deceased: 18.2%; *F*_1,2308_=2.474; *P*=.12); ICU admission (6.8% vs 7.3%; *F*_1,2308_=0.003; *P*=.96); or admission date (March 27, 2020, ± 8.3 days, vs March 28, 2020, ± 11.6 days). However, there were significant differences in terms of sex (37.2% women vs 42.2% women; *F*_1,2308_=5.768; *P*=.02) and days in hospital (mean 9, SD 6, vs mean 8, SD 7; *F*_1,2308_=4.786; *P*=.03). Notwithstanding, the effect size was small for both differences (η^2^=0.003 and η^2^=0.002, respectively).

**Table 1:** Laboratory tests used to characterize the patients.

Code	Description	Unit
RDW	Red cell distribution width	%
BAS	Basophils	×10^3^/µL
BAS%	Percentage of basophils	%
MCHC	Mean corpuscular hemoglobin concentration	g/dL
CREA	Creatinine	mg/dL
EOS	Eosinophils	×10^3^/µL
EOS%	Percentage of eosinophils	%
GLU	Glucose	mg/dL
AST	Aspartate transaminase	U/L
ALT	Alanine transaminase	U/L
MCH	Mean corpuscular hemoglobin	pg
HCT	Hematocrit	%
RBC	Red blood cells	×10^6^/µL
HB	Hemoglobin	g/dL
K	Potassium	mmol/L
LDH	Lactate dehydrogenase	U/L
LEUC	Leucocytes	×10^3^/µL
LYM	Lymphocytes	×10^3^/µL
LYM%	Percentage of lymphocytes	%
MONO	Monocytes	×10^3^/µL
MONO%	Percentage of monocytes	%
NA	Sodium	mmol/L
NEU	Neutrophils	×10^3^/µL
NEU%	Percentage of neutrophils	%
CRP	C-reactive protein	mg/L
PLAT	Platelet count	×10^3^/µL
BUN	Blood urea nitrogen	mg/dL
MCV	Mean cell volume	fL
MPV	Mean platelet volume	fL

### Clustering

Unsupervised automatic x-means clustering [32]—the implementation in RapidMiner Studio 9.7, Community Edition (RapidMiner, Inc)—was applied to the preprocessed data set that was previously described (see Data Preprocessing section). The algorithm determines the optimum number of clusters so that the intracluster distance of patients is at a minimum, and the intercluster distance of patients is at a maximum. The x-means algorithm was used instead of the more common k-means algorithm to overcome the three major shortcomings of the latter [32]: poor computational scaling, manual selection of the number of clusters, and tendency to local minima. X-means clustering determines the optimal number of clusters by the Bayesian information criterion (BIC), also known as the Schwarz criterion, which is used to maximize the explained variance by the clusters and minimize the number of parameters (k) [32]. X-means clustering is also an improvement over k-means clustering since it tends to create clusters formed by only one sample to minimize inertia [32]. Moreover, the later use of the Davies Bouldin index to evaluate the cluster distributions is also intended to overcome this issue since it considers a mix of both inertia and distortion to quantitatively asses the cluster models (see below). In addition, the automatic selection of the number of clusters by x-means clustering avoids the possible bias in the manual selection of k [32]. This bias is also present in hierarchical agglomerative clustering, where a threshold must be set to obtain the ultimate clusters after the hierarchy is built. Despite the fact that x-means clustering is not completely deterministic, it is certainly very stable with minimum variations between different runs [32] and is significantly more stable than k-means clustering. However, x-means clustering introduces a bias. Since it uses the BIC to evaluate the cluster models in each iteration, this criterion purposely favors the models with a lower number of clusters. This means that an alternative cluster model with a better Davies Boulding index and a higher number of clusters may have been discarded. However, a higher number of clusters with a better Davies Boulding index usually implies clusters with small numbers of samples—notice that the best index would be obtained by a model of one cluster per sample—which is not desirable at all for the clinical stratification purpose aimed for in this study.

Patients were considered here as vectors with several dimensions equal to the number of variables. In this case, the number of variables taken to apply the clustering algorithm was 842. None of the eight variables about demographics, hospital stay, and outcome measures were included. They were removed from the clustering formation because of the potential ethical controversies and biases (ie, demographics) or prospective information (ie, hospitalization stay and outcome measures). The algorithm was applied using several similarity or distance metrics between patients [33]: the Euclidean distance, the Canberra distance, the Chebyshev distance, the correlation similarity, the cosine similarity, the Dice similarity, the inner product similarity, the Jaccard similarity, the kernel Euclidean distance, the Manhattan distance, the max product similarity, the overlap similarity, the generalized divergence, the Itakura-Saito distance, the Kullback-Leibler divergence, the logarithmic loss, the logistic loss, the Mahalanobis distance, the squared Euclidean distance, and the squared loss. In spite of the fact that we could have had good similarity measure candidates a priori, based on data set characteristics such as dimensionality, the best practice was the selection based on empirical evaluation [34]. To avoid any a priori biases, we empirically tested all measures available in the software and kept the one yielding the best results.

To assess the fitness of the cluster distributions from the algorithm executions with the above metrics, the Davies Bouldin index was calculated for each one of them [35]. The Davies Bouldin index is a common measure that evaluates cluster models [35]. It quantifies the average maximum ratio of the within-cluster scatter to the between-cluster separation for every pair of clusters in a cluster model [35]. In other words, it provides a trade-off between intercluster similarity and intracluster distance [35]. With this definition, the lower the Davies Bouldin index the lower the within-cluster scatter and the higher the between-cluster separation, which is the most desirable property of a cluster model [35]. The Davies Bouldin index allowed us to quantitatively select the best cluster model among those created, one for each similarity measure considered.

### Cluster Validation

From the 1457 patients excluded due to missing values (ie, not used to obtain the clusters), we performed a validation analysis with the patients who presented no missing values in the variables that statistically differed between the three clusters obtained. Subsequently, these patients were assigned to one of the clusters previously obtained by using the best distance metric determined in the clustering process described above.

### Statistical Analysis

The difference in the 850 variables between all the clusters obtained was tested using a one-way multivariate analysis of variance. Pairwise post hoc comparisons between clusters were analyzed by the Bonferroni test. Significance was accepted at the 5% level (α=.05). The observed power and effect size, as partial η^2^, were reported for statistically significant differences.

## Results

Table 2 shows the number of clusters and the corresponding David Bouldin index of the cluster distribution of patients obtained by the x-means clustering algorithm for each of the similarity measures tested. Note that the lower the David Bouldin index, the better the cluster distribution (ie, higher intercluster distance and lower intracluster distance). The best cluster distribution (ie, lowest David Bouldin index) was obtained by using the Manhattan distance, which grouped the patients into three clusters.

**Table 2:** Number of clusters and the corresponding David Bouldin index.

Similarity measure	David Bouldin index	Number of clusters
Euclidean distance	0.948	3
Canberra distance	N/A^a^	1
Chebyshev distance	0.966	3
Correlation similarity	1.400	3
Cosine similarity	1.629	3
Dice similarity	N/A	1
Inner product similarity	N/A	1
Jaccard similarity	1.387	3
Kernel Euclidean distance	1.440	3
Manhattan distance	0.701	3
Max product similarity	N/A	1
Overlap similarity	5.099	4
Generalized divergence	3.445	3
Itakura-Saito distance	5.919	4
Kullback-Leibler divergence	5.677	4
Logarithmic loss	4.595	4
Logistic loss	3.445	3
Mahalanobis distance	4.595	4
Squared Euclidean distance	3.445	3
Squared loss	3.659	3

^a^N/A: not applicable; the David Bouldin index could not be calculated for these measures because they only had one cluster each.

Demographic and clinical characteristics of the patients in the three clusters are shown in Table 3. Notice that this table also shows the values of the eight variables (ie, demographics, hospital stay, and outcome measures) that were not used in the construction of the clusters (marked with a footnote in Table 3). Cluster 1 had a significantly higher proportion of deceased patients (46.6%) than cluster 2 (18.0%) and cluster 3 (10.5%). No significant difference in the percentage of ICU admissions was found between clusters. However, the patients who were admitted to the ICU in cluster 1 stayed a significantly shorter time than patients in cluster 3. No significant difference in sex was found between clusters. Patients in cluster 3 were significantly younger than those in cluster 1. In addition, patients in clusters 1 and 2 presented with a significantly higher heart rate in the emergency department than those in cluster 3. The average oxygen saturation for patients in the emergency department was significantly different between all clusters, whereby patients in cluster 1 had the lowest oxygen saturation and those in cluster 3 had the highest. With respect to previous diseases and surgical procedures, cluster 1 patients presented with a significantly higher percentage of epilepsy and emphysema than those in clusters 2 and 3. In addition, cluster 2 patients presented with a higher percentage of previous surgical procedures, as well as previous thoracic, thoracolumbar, and lumbosacral intervertebral disc disorders, than patients in cluster 3. Cluster 2 patients also presented with a significantly lower percentage of disorders of purine and pyrimidine metabolism than those in clusters 1 and 3. Finally, the percentage of patients who underwent surgical operations during their hospitalization was significantly higher in cluster 1 than in clusters 2 and 3.

Regarding laboratory tests, patients in cluster 1 showed significantly higher levels of serum creatinine, potassium, and blood urea nitrogen than those in clusters 2 and 3; cluster 1 patients also had a significantly higher value of red cell distribution width than did cluster 2 patients. In addition, patients in cluster 2 presented with significantly higher values of lymphocytes and serum levels of sodium, and significantly lower platelet counts than patients in cluster 3. In addition, cluster 3 patients showed lower values of mean corpuscular hemoglobin concentration and leucocytes, serum levels of alanine transaminase (ALT), and percentage of neutrophils than did patients in clusters 1 and 2. Cluster 3 patients had significantly higher values and percentages of eosinophils and percentages of lymphocytes than did patients in clusters 1 and 2. Finally, the laboratory tests that showed significant differences between all clusters were found for the serum levels of aspartate transaminase (AST) (cluster 1 > cluster 2 > cluster 3), lactate dehydrogenase (LDH) (cluster 1 > cluster 2 > cluster 3), C-reactive protein (CRP) (cluster 1 > cluster 2 > cluster 3), and the number of neutrophils (cluster 1 > cluster 2 > cluster 3).

**Table 3:** Demographic and clinical characteristics of patients (N=853) in the three clusters.

Characteristics			Cluster 1 (n=58)	Cluster 2 (n=300)	Cluster 3 (n=495)	*F* test (*df*=2, 850)		*P* value			η^2a^			1–β^b^		
**Demographics**																
	Age (years)^c^, mean (SD)		71.1 (13.7)^d^	67.0 (15.1)^d,e^	65.1 (16.2)^e^	3.457		.03			0.009			0.648		
	Sex (men)^c^, n (%)		41 (70.7)^d^	181 (60.3)^d^	313 (63.3)^d^	1.027		.36			0.003			0.23		
**Hospital stay and outcome measures**																
	Inpatient hospital days^c^, mean (SD)		8.5 (4.9)^d^	8.6 (6.4)^d^	8.3 (5.1)^d^	0.363		.70			0.001			0.109		
	**Discharge reason^c^, n (%)**						26.054		<.001			0.062			1	
		Recovered	31 (53.4)^d^	246 (82.0)^e^	443 (89.5)^e^											
		Deceased	27 (46.6)^d^	54 (18.0)^e^	52 (10.5)^e^											
	**Intensive care unit admission^c^, n (%)**						1.12		.33			0.003			0.248	
		No	52 (89.7)^d^	277 (92.3)^d^	458 (92.5)^d^											
		Yes	6 (10.3)^d^	23 (7.7)^d^	37 (7.5)^d^											
	Days until intensive care unit admission^c^, mean (SD)		0.2 (0.4)^d^	3.4 (6.3)^d^	2.3 (4.3)^d^	1.393		.26			0.042			0.289		
	Days in intensive care unit^c^, mean (SD)		0.2 (0.4)^d^	4.8 (6.5)^d,e^	7.6 (6.9)^e^	3.747		.03			0.106			0.665		
	Mechanical ventilation need^c^, n (%)		35 (60.3)^d^	177 (59.0)^d^	277 (56.0)^d^	0.163		.85			<0.001			0.075		
**Vital signs and laboratory tests, mean (SD)**																
	First heart ratio measurement in the emergency department		98.4 (25.0)^d,e^	100.1 (26.2)^d^	93.5 (24.4)^e^	8.45		<.001			0.021			0.965		
	First oxygen saturation measurement in the emergency department		84.2 (12.3)^d^	90.1 (7.6)^e^	94.2 (3.6)^f^	81.732		<.001			0.171			1		
	Last heart ratio measurement in the emergency department		99.0 (25.1)^d,e^	100.1 (26.0)^d^	93.6 (24.7)^e^	8.104		<.001			0.02			0.958		
	Last oxygen saturation measurement in the emergency department		84.2 (12.2)^d^	90.0 (7.52)^e^	94.2 (3.6)^f^	82.554		<.001			0.172			1		
	Red cell distribution width (%)		13.6 (1.9)^d^	12.9 (1.84)^e^	13.0 (1.9)^d,e^	3.28		.04			0.008			0.623		
	Basophils (×10^3^/µL)		0.03 (0.03)^d^	0.02 (0.02)^d,e^	0.02 (0.0)^e^	5.545		.004			0.014			0.854		
	Mean corpuscular hemoglobin concentration (g/dL)		33.9 (1.5)^d^	34.0 (1.17)^d^	33.6 (1.2)^e^	8.602		<.001			0.021			0.968		
	Creatinine (mg/dL)		1.3 (1.4)^d^	1.0 (0.47)^e^	1.0 (0.5)^e^	9.591		<.001			0.024			0.981		
	Eosinophils (×10^3^/µL)		0.02 (0.04)^d^	0.02 (0.04)^d^	0.04 (0.1)^e^	6.518		.002			0.016			0.908		
	Eosinophils (%)		0.20 (0.5)^d^	0.3 (0.60)^d^	0.6 (1.2)^e^	10.000		<.001			0.025			0.985		
	Aspartate transaminase (U/L)		80.3 (48.0)^d^	55.8 (33.4)^e^	32.8 (18.7)^f^	109.193		<.001			0.216			1		
	Alanine transaminase (U/L)		57.2 (69.1)^d^	50.7 (48.1)^d^	29.5 (23.8)^e^	32.686		<.001			0.076			1		
	Potassium (mmol/L)		4.6 (0.8)^d^	4.2 (0.6)^e^	4.2 (0.5)^e^	16.957		<.001			0.041			1		
	Lactate dehydrogenase (U/L)		1339.72 (240.56)^d^	742.5 (122.0)^e^	447.7 (91.5)^f^	1666.635		<.001			0.808			1		
	Leucocytes (×10^3^/µL)		9.9 (4.8)^d^	8.5 (4.2)^d^	6.9 (5.2)^e^	13.055		<.001			0.032			0.997		
	Lymphocytes (×10^3^/µL)		1.0 (0.5)^d,e^	1.0 (0.6)^d^	1.3 (2.1)^e^	3.692		.03			0.009			0.679		
	Lymphocytes (%)		12.6 (7.8)^d^	14.0 (7.7)^d^	20.0 (9.8)^e^	46.962		<.001			0.106			1		
	Monocytes (%)		5.1 (2.9)^d^	6.6 (3.9)^d^	8.7 (4.8)^e^	29.321		<.001			0.069			1		
	Sodium (mmol/L)		136.2 (7.1)^d,e^	136.2 (4.4)^d^	137.2 (4.6)^e^	4.016		.02			0.01			0.718		
	Neutrophils (×10^3^/µL)		8.4 (4.7)^d^	6.9 (4.0)^e^	4.9 (2.7)^f^	45.584		<.001			0.103			1		
	Neutrophils (%)		81.8 (10.2)^d^	78.8 (9.9)^d^	70.4 (11.9)^e^	62.070		<.001			0.135			1		
	C-reactive protein (mg/L)		206.1 (131.7)^d^	152.1 (110.0)^e^	64.2 (63.7)^f^	12.930		<.001			0.223			1		
	Platelet count (×10^3^/µL)		229.0 (92.2)^d,e^	236.3 (96.6)^d^	210.3 (87.2)^e^	7.541		.001			0.019			0.944		
	Blood urea nitrogen (mg/dL)		58.9 (56.6)^d^	41.8 (29.0)^e^	40.5 (29.7)^e^	7.579		.001			0.019			0.945		
**Diseases and surgical procedures, n (%)**																
	Previous history of disorders of purine and pyrimidine metabolism		4 (6.9)^d^	4 (1.3)^e^	25 (5.1)^d^	4.179		.02			0.01			0.736		
	Previous history of epilepsy and recurrent seizures		3 (5.2)^d^	4 (1.3)^e^	2 (0.4)^e^	5.660		.004			0.014			0.862		
	Previous history of emphysema		3 (5.2)^d^	2 (0.7)^e^	2 (0.4)^e^	6.663		.001			0.017			0.914		
	Previous history of thoracic, thoracolumbar, and lumbosacral intervertebral disc disorders		0 (0)^d,e^	9 (3.0)^d^	3 (0.6)^e^	4.385		.01			0.011			0.758		
	Previous history of surgical procedures		0 (0)^d,e^	4 (1.3)^d^	0 (0)^e^	3.753		.02			0.009			0.686		
	Surgical operations during the current hospitalization		3 (5.2)^d^	2 (0.7)^e^	4 (0.8)^e^	4.880		.008			0.012			0.804		

^a^Effect size.

^b^Observed power.

^c^These variables were not used for the cluster construction.

^d-f^Values in the same row, but in different columns, that do not share footnote letters were significantly different after Bonferroni post hoc correction; values in the same row, but in different columns, that share footnote letters were not significantly different.

**Figure 1:**
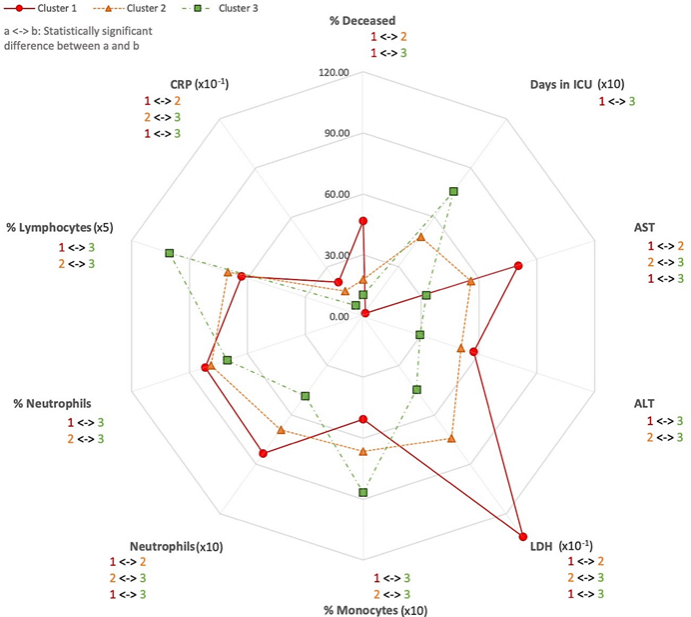
Hospital stay, outcome measures, and laboratory tests that showed statistically significant differences among clusters with a medium or high effect size (η^2^>0.06). Note that some variables are scaled (transformation between brackets) for the sake of graph legibility. ALT: alanine transaminase; AST: aspartate transaminase; CRP: C-reactive protein; ICU: intensive care unit; LDH: lactate dehydrogenase.

For a clearer characterization of the clusters, Figure 1 shows a radar chart with the variables (ie, hospital stay, outcome measures, and laboratory tests) that showed statistically significant differences among the clusters and a medium or high effect size (η^2^>0.06) [36].

A web-based cluster assignment tool, based on the results reported here, can be found online [37].

To test the robustness of the identified clusters, we performed a validation analysis using the initially excluded patients who did not have missing values in the variables that statistically differed among the three clusters (Table 3). Specifically, it was based on six variables (ie, first and last oxygen saturation measurement in the emergency department, AST, LDH, neutrophils, and CRP). For this purpose, we selected 349 patients who were initially excluded and who were assigned to one of the three previously identified clusters by the minimum Manhattan distance to the average values of the six mentioned variables of those clusters. Table 4 shows the differences in demographics, hospital stay, and outcome measures in the three clusters. Indeed, the clusters initially obtained were consistent with the clusters assigned in the validation analysis in terms of age, sex, hospital stay, and outcome measures. Specifically, cluster 1 was the one with the oldest and with the highest proportion of deceased patients. By contrast, cluster 3 was the one with the youngest and with the lowest proportion of deceased patients.

**Table 4:** Demographics as well as hospital stay and prognosis of the patients (n=349) selected for the validation analysis in the three clusters.

Characteristics				Cluster 1 (n=18)		Cluster 2 (n=112)		Cluster 3 (n=219)		*F* test (*df*)		*P* value			η^2a^			1–β^b^	
**Demographics**																			
	Age (years), mean (SD)		72.8 (14.2)^c,d^		71.3 (14.3)^c^		64.2 (15.8)^d^		9.414 (2, 346)		<.001			0.052			0.979		
	Sex (men), n (%)		14 (77.8)^c^		68 (60.7)^c^		123 (56.2)^c^		1.738 (2, 346)		.18			0.01			0.364		
**Hospital stay and outcome measures**																			
	Inpatient hospital days, mean (SD)		9.1 (6.4)^c^		9.3 (5.9)^c^		8.0 (5.3)^c^		2.320 (2, 346)		.10			0.013			0.469		
	**Discharge reason, n (%)**									22.025 (2, 346)		<.001			0.113			1	
		Recovered	8 (44.4)^c^		80 (71.4)^d^		200 (91.3)^e^												
		Deceased	10 (55.6)^c^		32 (28.6)^d^		19 (8.7)^e^												
	**Intensive care unit admission, n (%)**									4.268 (2, 346)		.02			0.024			0.743	
		No	16 (88.9)^c,d^		101 (90.2)^c^		213 (97.3)^d^												
		Yes	2 (11.1)^c,d^		11 (9.8)^c^		6 (2.7)^d^												
	Days until intensive care unit admission, mean (SD)		6.5 (7.8)^c^		4.1 (3.9)^c^		6.3 (13.7)^c^		0.170 (2,16)		.84			0.021			0.072		
	Days in intensive care unit, mean (SD)		4.5 (0.7)^c^		3.8 (4.5)^c^		3.2 (4.6)^c^		0.082 (2,16)		.92			0.01			0.06		
	Mechanical ventilation need, n (%)		12 (66.7)^c^		54 (48.2)^c^		96 (43.8)^c^		1.854 (2, 346)		.16			0.011			0.385		

^a^Effect size.

^b^Observed power.

^c-e^Values in the same row, but in different columns, that do not share footnote letters were significantly different after Bonferroni post hoc correction; values in the same row, but in different columns, that share footnote letters were not significantly different.

## Discussion

With the application of an unsupervised machine learning approach, we could identify and segregate patients with COVID-19 into subgroups depending on the severity of disease, simply by using standard laboratory tests performed during the first medical assessment in the emergency department. We found that inflammatory (ie, CRP), hematologic (ie, number of neutrophils and percentage of monocytes and lymphocytes), and serum biochemical abnormalities (ie, AST, ALT, and LDH), mainly indicating liver dysfunction, detected upon admission to the hospital could predict the severity of the disease. From a sum of 850 variables collected in the emergency department, only four standard laboratory tests (ie, serum levels of AST, LDH, CRP, and the number of neutrophils) were enough to segregate these patients into three separate clusters. Of these, the levels of LDH had the biggest effect size, practically allowing us to differentiate the three clusters linearly. Further, the percentage of monocytes and lymphocytes as well as ALT distinguished cluster 3 patients (ie, less severe) from patients in the other two clusters. Cluster 1 was characterized by the highest proportion of deceased patients; the highest levels of AST, ALT, LDH and CRP; the highest number of neutrophils; and the lowest percentages of monocytes and lymphocytes (Figure 1). Cluster 2 included a lower proportion of deceased patients and intermediate values of the previous laboratory tests (Figure 1). Finally, the lowest proportion of deceased patients; the lowest levels of AST, ALT, LDH and CRP; the lowest number of neutrophils; and the highest percentages of monocytes and lymphocytes characterized cluster 3 (Figure 1).

Our results have several clinical implications. First, age and sex were not considered while building the clusters. Therefore, our unsupervised machine learning approach, based exclusively on the performance of simple laboratory tests at a primordial stage, would permit the establishment of a strategy for rationing of health care resources and to establish a triage protocol, which would support medical decisions in a transparent and ethical way. Second, since the analyzed data are from standard laboratory tests, this method would be especially valuable for underdeveloped and developing regions that lack medical resources and have affordability issues. Finally, we could tailor treatment to each severity group accordingly at a primordial stage (ie, in the emergency department). For example, more aggressive therapies could be considered in patients classified in cluster 1 (ie, the most severe) and not in those in cluster 3 (ie, the least severe).

Initially, SARS-CoV-2 was primarily considered a respiratory pathogen. However, with time, it has behaved like a virus with the potential to cause multisystem involvement [38,39]. Specifically, hepatic injury related to COVID-19 is only beginning to unravel. Elevated liver injury indicators, particularly AST, are strongly associated with a higher mortality risk in patients with COVID-19 [40]. Of note, high serum levels of LDH predict higher in-hospital mortality in patients with severe and critical condition of COVID-19 [41]. Significant increased CRP levels in the early stages of COVID-19 are correlated with the severity of disease and the degree of internal tissue pathologies [42]. Further, a significant increase in the number of neutrophils with a decrease in the number of lymphocytes, monocytes, and eosinophils may indicate clinical worsening and increased risk of a poor outcome among patients with COVID-19 [43]. Taken together, the presence of elevated biomarkers of inflammation and that of liver injury in serum, as well as the number of neutrophils at admission, are indicative of multiple organ failure in patients with COVID-19 that could lead to death. Our laboratory findings are in agreement with other previous studies worldwide [44-46].

Although one previous multicenter study, based on the analyses of demographics, comorbidities, vital signs, and laboratory test results upon admission, that evaluated the prediction of disease course in patients with COVID-19 has been undertaken [18], there remains much to learn about applying machine learning techniques regarding this novel infectious disease. Comparison with that study is difficult, as they had used different variables and techniques. The accuracy of the model could be influenced by several factors, including the methods. Feature extraction methods, feature selection or classification tools, number of subjects, and demographics are also important considerations. Besides, most COVID-19 diagnostics and prognostic models that have evolved to date have a high risk of generating bias leading to inequality [47], mainly due to the high influence of demographic variables, especially age and sex, in those models and to the nonblinded nature of the supervised machine learning approach between predictors and outcome measures. In fact, our results confirmed that age and sex had a similar and low discriminant value to separate the three clusters (Table 3). Nevertheless, the results obtained in our study are in line with most previous work based on supervised machine learning techniques in COVID-19 [18,47].

The study should be interpreted within the context of several limitations. First, the patients in this study may represent a selected group of patients with COVID-19 (ie, patients with a more severe disease, since all of them were admitted to the hospital); hence, it is questionable as to what extent our results could be generalized to the entire population of patients with COVID-19. The reason for this was that the extreme circumstances in our hospitals at the peak of this pandemic permitted the hospitalization of only the most severe cases. Notwithstanding, our aim was to detect severity subgroups among patients with COVID-19 upon admission to the hospital. Second, we only kept the records (ie, patients), laboratory tests, and clinical variables from 853 patients from the data set due to the high number of missing values in the remaining 1457 patients. Despite this, the results have been robust.

In closing, to the authors’ knowledge, the work presented in this paper is the first attempt to use unsupervised machine learning to identify severity subgroups among patients with COVID-19 upon admission. A few affordable, simple, and standard laboratory tests, which are expected to be available in any emergency department, have shown promising discriminative power for characterization of severity subgroups among patients with COVID-19. We have also provided an online severity cluster assignment tool for patients with COVID-19 who are admitted to the emergency department [37]. This could permit the classification of patients according to severity subgroups and, hence, initiate earlier interventions.

## Abbreviations

ALT: alanine transaminase
AST: aspartate transaminase
BIC: Bayesian information criterion
CRP: C-reactive protein
ICD-10: International Statistical Classification of Diseases and Related Health Problems, 10th Revision
ICU: intensive care unit
LDH: lactate dehydrogenase
